# Healthcare and School Professionals’ Satisfaction with Implementation of Finnish Smart Family Practice in Poland

**DOI:** 10.3390/pediatric17060120

**Published:** 2025-11-05

**Authors:** Justyna Nowak, Agata Szymczak, Marta Morawska, Heli Kuusipalo, Emma Koivurinta, Kati Kuisma, Päivi Mäki, Taina Sainio, Nella Savolainen, Katarzyna Brukało

**Affiliations:** 1 Department of Cardiovascular Disease Prevention, Department of Metabolic Disease Prevention, Faculty of Public Health in Bytom, Medical University of Silesia, 41-900 Bytom, Poland; 2 Office of Public Partnership and Innovation, National Health Fund, Central Office, 02-528 Warsaw, Poland; agata.szymczak@nfz.gov.pl; 3 Department of Analysis, Quality Monitoring and Optimization of Benefits, National Health Fund, Central Office, 00-863 Warsaw, Poland; 4 Finnish Institute for Health and Welfare, 00271 Helsinki, Finland; 5 Finnish Heart Association, 00621 Helsinki, Finland; 6 Department of Health Policy, Faculty of Public Health in Bytom, Medical University of Silesia, 41-902 Bytom, Poland

**Keywords:** Smart Family Practice, lifestyle change interventions, professional competencies, strategies for obesity treatment

## Abstract

**Background:** Poland is one of six countries implementing the Finnish Smart Family practice under the Joint Action Health4EUKids, aimed at supporting families in adopting lifestyle counseling methods and preventing childhood obesity across the European Union. **Material and method:** Since March 2024, Poland has implemented Smart Family tools through training sessions for professionals who work or will work with families of children with excess body weight. A total of 295 individuals have been trained, including 52.2% dietitians, 34.9% nurses, and 12.9% school staff such as teachers, school counselors, and psychologists. Before and after the training sessions, participants completed a survey assessing their knowledge of the Smart Family Practice, and familiarity with supportive tools. **Results:** Among 295 participants, nearly half reported no prior experience with family-based lifestyle change interventions. Post-training, over 70% expressed readiness to implement the SMART FAMILY method, with high interest (80.7%), motivation (76.5%), and satisfaction (83.6%). Most recognized its potential to support healthy lifestyles and parental engagement (>85%). Key barriers included lack of family cooperation (87.8%), staff shortages (81.0%), limited training (78.4%), and insufficient resources (43%). **Conclusions:** A high level of acceptance and motivation among participants indicates that the SMART FAMILY method has the potential for effective adaptation in Poland. Its implementation requires strengthening specialists’ skills and providing appropriate organizational resources. Overcoming barriers such as lack of experience, limited time, and difficulties in engaging families is crucial to achieving lasting intervention outcomes.

## 1. Introduction

Over the past three decades, excessive body weight—encompassing overweight and obesity—has emerged as one of the most serious public health challenges worldwide. Between 1990 and 2021, the proportion of children and adolescents affected by overweight and obesity doubled, while the prevalence of obesity alone tripled. By 2021, obesity affected more than 93 million children aged 5–14 years and over 80 million individuals aged 15–24 years [1,2]. This global trend poses a major burden on healthcare systems and has far-reaching implications for individual health and societal well-being.

Europe is considered a high-risk region for childhood overweight and obesity, with notable regional differences. In Western and Northern Europe, a growing shift from overweight to obesity dominance has been observed. Projections suggest that if current trends persist, by 2050 obesity prevalence in children aged 5–14 years will exceed overweight prevalence, leading to significant health and social consequences. Data from the World Health Organization (WHO) Childhood Obesity Surveillance Initiative (COSI, 2015–2017) indicate that southern European countries exhibit the highest rates of childhood obesity, with prevalence ranging from 18% to 21% among boys in Cyprus, Greece, Italy, Malta, San Marino, and Spain. In contrast, Denmark, France, Ireland, Latvia, and Norway report the lowest rates, ranging from 5% to 9% for either sex [3,4]. These differences highlight the importance of context-specific prevention and intervention strategies.

In Poland, overweight and obesity among children and adolescents represent a growing public health problem. Over the past two decades, this issue has intensified, particularly among school-aged boys. Projections indicate that by 2050 the proportion of children with obesity will surpass those with overweight. After a decade in which Poland’s prevalence rates were below the European average, current figures now approach levels observed in Western European countries. Childhood overweight and obesity are associated not only with an increased risk of diet-related diseases—such as type 2 diabetes, hypertension, and dyslipidemia—but also with psychological consequences, including low self-esteem, stigmatization, and reduced quality of life. National data show that overweight affects about 14% of girls and nearly 20% of boys in Poland. Among children and adolescents aged 7–18 years, prevalence ranges from 18.8% to 24.6% in boys and from 14.3% to 17.4% in girls. These trends have been steadily rising for over 40 years, and given that obese children are highly likely to become obese adults, untreated childhood obesity poses a significant threat to the sustainability of the healthcare system [5,6,7,8,9,10,11].

Addressing childhood obesity requires multidimensional, context-specific interventions that are sustained over time and integrated into the child’s environment. Effective strategies must target not only the child but also their family and broader community. Evidence indicates that interventions combining dietary modifications, increased physical activity, and psychological support—tailored to the individual needs of participants—are the most effective. These interventions should be long-term and delivered by interdisciplinary teams to ensure comprehensive care. Engaging both children and their families in structured, evidence-based programs fosters sustainable lifestyle changes, reduces obesity rates, and improves overall health outcomes [5,11].

A promising approach in Poland is the adaptation and implementation of the Finnish Smart Family Practice (Neuvokas perhe), initiated in December 2022 as part of the Joint Action Health4EUkids initiative. Coordinated by the National Health Fund and the Medical University of Silesia, this program represents the adaptation of an evidence-based model recognized by the European Commission as a best practice [12,13,14,15,16,17]. The Smart Family method supports families of children with non-normative body weight by integrating primary healthcare with environmental health determinants. Its core principles include active family engagement, motivation, positive communication, and the empowerment of participants to build on their strengths in nutrition, physical activity, sleep hygiene, and other health-related behaviors [12,13,14].

The program employs practical tools—including the Smart Family Card, Child’s Nutrition Habits Card, and Screen Time Card—enabling families to self-assess lifestyles, set individual goals, and actively participate in healthcare processes [13,18]. In Poland, eleven tools adapted from the Finnish model to the national context (including translations, terminology, and applied graphics), along with two additional tools developed by the Polish team, have been made available online, together with training and educational materials intended for primary healthcare teams [16,18]. The tools developed by the Polish team included the Family Shopping Card and the Habit Change Agreement Card. The Family Shopping Card supports families in planning healthy and organized grocery shopping by creating weekly shopping lists and establishing shared rules, promoting healthy eating, saving time and money, and enhancing family cooperation. The Habit Change Agreement Card helps families develop and maintain healthy habits through joint agreements on specific behaviors, fostering responsibility in children, strengthening family bonds, and encouraging a healthy lifestyle.

As part of its implementation in Poland, comprehensive training materials were prepared, and in-person training sessions were conducted in several cities for nurses, dietitians, and professionals working with children with obesity, such as teachers, school counselors, and psychologists [17]. Educational resources for primary healthcare teams and other specialists were also developed, including an e-learning course consisting of four modules: Smart Family tools, motivation and positive communication, principles of nutrition for school-aged children, and physical activity, sleep, and leisure time [19]. Additionally, a practical guide entitled “How to Talk about Childhood Obesity? A Guide for Professionals Working with Families in the Healthy Family Program” was developed. Intervention activities in selected primary healthcare facilities facilitated the direct implementation of the program and allowed preliminary evaluation of its effectiveness in practice [20].

The aim of this study is to assess the level of satisfaction and identify factors influencing the implementation of the Finnish Smart Family Practice in Poland from the perspective of healthcare and school professionals who participated in the training, including dietitians, nurses, teachers, school counselors, and psychologists.

## 2. Materials and Methods

**Study Design and Participants**

Since November 2023, the Smart Family program has been implemented in Poland to support families of children with excess body weight. The program targeted professionals working with these families, including dietitians, nurses, and school staff (teachers, school counselors, and psychologists). The inclusion criteria for this study encompassed individuals who met at least one of the following conditions: being a final-year (fifth year) nursing student, being a final-year master’s student in dietetics, possessing formal education in dietetics, participation in a conference organized by the Polish Society of Dietetics, or being a teacher, school counselor, or school psychologist. Participant selection was purposive (non-random) and targeted individuals actively working or training in the fields of healthcare and education, with direct professional contact with families of children with overweight or obesity. Exclusion criteria included the absence of written informed consent to participate in the study, failure to meet any of the inclusion criteria, and not performing or not planning to perform professional work in the field of healthcare or school education in which the recipient of services would be a family with an overweight or obese child.

According to Polish law, this study did not qualify as a medical experiment and therefore did not require approval from the Bioethics Committee (Act of 5 December 1996 on the Professions of Physician and Dentist, Journal of Laws 2024, item 1287, as amended).

A total of 295 individuals participated in the training sessions: 52.2% dietitians, 34.9% nurses, and 12.9% school staff. Participants completed surveys before and after the training to assess their knowledge of the Smart Family method, skills in motivational interviewing, and familiarity with supportive tools. Each participant provided informed written consent to take part in the training.

**Description of the Training**

The training was conducted according to a detailed agenda, ensuring consistency across all sessions. It began with a discussion of the Finnish Smart Family practice, including its objectives, the fundamental concept of positive development, as well as the main target groups and areas of application. The subsequent part of the training focused on the role of motivation in changes to family lifestyle. Participants were introduced to motivation in the context of dietary behavior change, the transtheoretical model of behavior change, and strategies for working with families at different stages of this process, as well as various types of motivation (external, internal, positive, and negative). Practical strategies for goal-setting using the GROW and SMART models were presented, alongside the pillars of motivation and the application of motivational interviewing in supporting family lifestyle changes.

A central element of the training involved the presentation and practical exercises using the Smart Family tools, such as the Healthy Family Card (covering children’s and parents’ dietary and physical activity habits), the Star of Our Family, the Family Screen Time Agreement, and the “Sleep Tree” exercise [13,16]. The training adopted an interactive approach, encouraging participants to engage in discussions, share experiences and reflections on the method, its strengths and weaknesses, as well as potential barriers to its implementation.

**Training Procedure and Participant Evaluation**

All participants were trained under identical conditions, using the same standardized materials and led by the same instructor to ensure consistency and methodological rigor. At the beginning of each session, participants were introduced to the objectives of the training and provided informed written consent to participate. The training was conducted in a structured format, combining theoretical instruction with a discussion of the method and a presentation of Smart Family tools, aiming to increase engagement and facilitate skill acquisition.

The effectiveness of the training was assessed using questionnaires completed before and after the sessions. At the start of the training, participants completed a survey that included questions on: (1) their prior experience working on lifestyle change interventions with entire families, (2) their self-assessed knowledge of the Smart Family practice, (3) their self-rated ability to apply motivational interviewing techniques with families, and (4) familiarity with tools supporting work with families of children with overweight or obesity. After completing the training, participants filled in evaluation questionnaire. The Implementation Motivation Questionnaire assessed their motivation, possible barriers to using the Smart Family method in everyday practice, and their confidence in applying it. Together, these two tools provided a broad assessment of both participants’ motivation and the practical skills gained during the training.

**Statistical methods**

The results are presented as percentages (%) and the number of responses (N) for each category. Data were analyzed descriptively, taking into account the frequency of individual variables among the participants. All data were processed and presented using standard tables and figures.

## 3. Results

**Preliminary Self-Assessment of Competencies in Lifestyle Change Interventions within the Smart Family Program**

**Figure 1:**
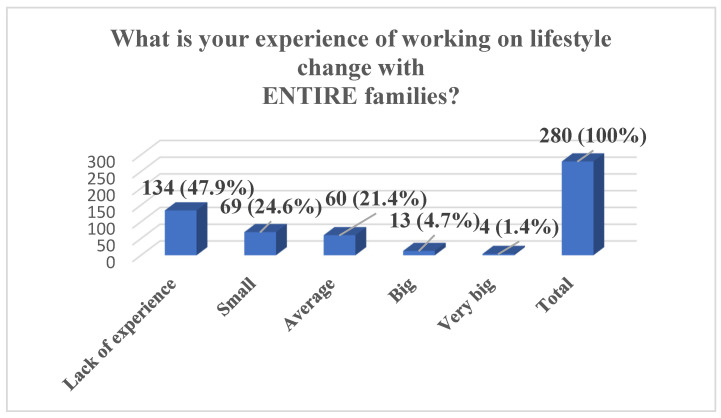
Participants’ Experience of Working on Lifestyle Change with Entire Families, *n* (%).

**Figure 2:**
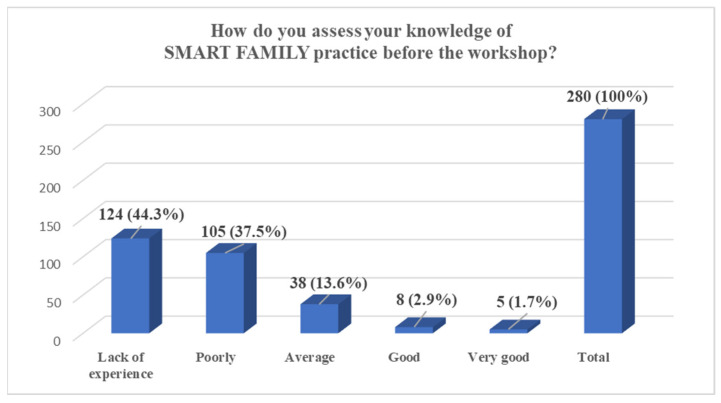
Self-Assessment of Knowledge on SMART FAMILY Practice Before the Workshop, *n* (%).

**Figure 3:**
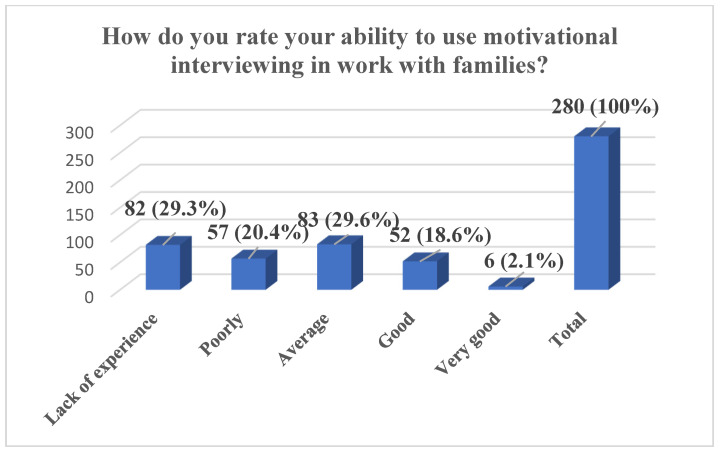
Self-Rated Ability to Use Motivational Interviewing in Work with Families, *n* (%).

**Figure 4:**
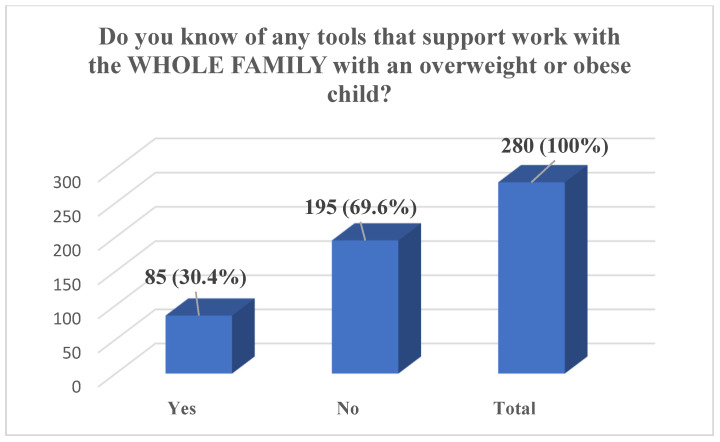
Awareness of Tools Supporting Work with the Whole Family of an Overweight or Obese Child, *n* (%).

In total, during the entire implementation period of the SMART FAMILY practice, 295 individuals were trained, of whom 280 completed the full pre-training questionnaire. The results show that nearly half of the participants (47.9%) reported having no experience in working on lifestyle change with entire families, while an additional 24.6% indicated only a small amount of experience. A further 21.4% assessed their experience as average, and only a small proportion reported having significant experience—4.7% as big and 1.4% as very big. The majority of participants also assessed their knowledge of SMART FAMILY practice before the workshop as limited: 44.3% reported a lack of experience and 37.5% rated their knowledge as poor. Only 13.6% considered their knowledge to be average, and a minority rated it as good (2.9%) or very good (1.7%). Regarding motivational interviewing, participants reported varied levels of self-assessed competence, with the largest group (29.6%) rating their ability as average and 29.3% reporting no experience. Additionally, 20.4% rated their skills as poor, 18.6% as good, and 2.1% as very good. Finally, a substantial majority of participants (69.6%) indicated that they were not aware of any tools to support work with the whole family of an overweight or obese child, with only 30.4% reporting familiarity with such tools (Figure 1, Figure 2, Figure 3 and Figure 4).

**Implementation Motivation Questionnaire: Participant Self-Assessment Results**

After completing the training, 235–238 of all participants completed the Implementation Motivation Questionnaire, providing self-assessment results regarding their experience, knowledge, and skills in applying the SMART FAMILY practice (Table 1). The analysis of the results indicates a high level of acceptance and positive attitudes among participants towards the training and the Smart Family method. The vast majority of respondents (80.7%) declared personal interest in the training topics, while 71.8% reported that, after familiarizing themselves with the materials, they would be able to implement the method in their professional practice. Furthermore, 76.5% expressed motivation to adopt Smart Family, and 83.6% were satisfied with their participation in the project, which was also reflected in their declared commitment to its implementation (66.1%). Respondents also highly assessed the potential impact of Smart Family on clients—85.8% agreed that the method supports a healthy lifestyle and self-efficacy, and 85.3% recognized its importance in encouraging parents to actively promote their children’s health-related behaviors. Additionally, 67.9% of participants perceived a positive influence of the method on their professional community. Opinions were more diverse regarding the conditions for implementation—58.7% considered the timing appropriate, while only 43% believed that their workplace had sufficient resources to support implementation.

The analysis of data on perceived barriers to implementing the Smart Family method (Table 2) shows that participants see several factors that may make implementation difficult. The most often mentioned obstacle was lack of cooperation with families—87.8% of respondents saw this as a potential barrier. Other common difficulties included lack of staff (81.0%), lack of own training in the method (78.4%), and lack of time (73.2%). Participants also noted lack of materials needed to carry out the program (70.6%), as well as lack of support from their direct supervisor (67.3%) and co-workers (65.7%). In addition, 63.5% of respondents indicated that existing routines could hinder implementation.

The analysis of results about participants’ confidence (Table 3) shows that most respondents have a moderate or high level of belief in their ability to implement the Smart Family method in their work. Regarding the question about changing their way of working according to the Smart Family model, 59.9% of participants said they were “fairly confident” or “completely confident,” with the largest group (33.3%) choosing “completely confident.” Similar results were found for the question about implementing the method in their work community—54.7% felt confident or fairly confident, with 29.8% reporting complete confidence. For the question about whether customers, families, and children would like the Smart Family method, responses were more mixed: 44.9% were fairly or completely confident, but the largest group (38.7%) answered “neither confident nor doubtful,” which may suggest a need for further evaluation of end-user acceptance.

## 4. Discussion

The preliminary self-assessment analysis of competencies among Smart Family Practice participants identified notable gaps in experience, knowledge, and resources required to implement lifestyle change interventions with entire families. The findings indicate that nearly half of the participants (47.9%) reported having no experience in working with families on lifestyle change, and an additional 24.6% described their experience as limited.

It is important to emphasize that the analysis concerned the pre-training stage—participants completed the questionnaire before the training began. Therefore, the fact that most participants reported no or limited experience and restricted knowledge of the Smart Family Practice is natural and expected. The pre-training questionnaire was designed to capture the starting point, allowing for the assessment of changes in competencies after the training.

The analysis of the results indicates that work in the domain of lifestyle change interventions involving entire families remains a marginal component of professional practice. This may stem both from the fact that a portion of the participants are still completing their education and from the lack of sufficient experience and preparation among professionals actively engaged in fields that involve interaction with families of children affected by overweight or obesity. This phenomenon is concerning, as it reveals a competency gap that may hinder the effectiveness of early intervention. Professionals working with such families should possess the requisite skills and knowledge to identify needs at an early stage and to propose the implementation of appropriate therapeutic or educational measures. Findings from a study conducted across four pediatric endocrinology centers in Poland (2022–2023) reveal a substantial delay in referring children with diagnosed obesity to specialist care—on average by approximately seven years from the point of diagnosis. This highlights the need for early intervention strategies that are tailored to the patient’s age, gender, and developmental stage [21].

The identified low level of participants’ knowledge regarding the SMART FAMILY practice prior to training (with 81.8% assessing their knowledge as insufficient or weak) unequivocally highlights the need for systematic education and support for specialists operating in this field. Another salient aspect is the lack of awareness of available tools to support work with entire families of children affected by overweight or obesity—with as many as 69.6% of participants reporting no familiarity with such resources. In response to this need, materials utilized within the Smart Family practice were developed and made freely accessible via the Polish project website [16]. Moreover, all training participants were enrolled in a mailing list through which they received regular newsletters, reinforcing engagement with the project and providing detailed information on the tools and their application. This approach facilitated sustained contact between participants and the Smart Family practice, as well as with the relevant tools and their implementation guidelines.

To ensure the durability of the project’s impact, an e-learning course comprising four modules was developed: Smart Family tools; the role of motivation and positive communication with families; principles of nutrition for school-aged children; and physical activity, sleep, and leisure time. The course is freely available on the National Health Fund’s website and may be utilized by any specialist working with children affected by obesity [17]. Additional support was provided through a professional brochure, “How to Talk About Childhood Obesity? A Guide for Specialists Working with Families in the Healthy Family Program”, offering practical recommendations for working with families confronting obesity [19].

Ensuring continuous access to information about the project fosters sustained engagement of participants in implementing the Smart Family practice in daily work. This constitutes a critical factor for consolidating training outcomes, enhancing specialists’ awareness and competencies, and thereby improving the effectiveness of interventions.

The obtained results demonstrate a high level of participant acceptance of the training and the Smart Family method, as evidenced both by expressed interest in the subject matter and by readiness to implement it in professional practice. Over 80% of respondents indicated personal interest in the topics addressed, and nearly three-quarters (71.8%) assessed that, after reviewing the materials, they possess the competencies required to apply the method in their work. These findings suggest that the intervention was perceived as both relevant and useful, thereby enhancing the likelihood of its practical implementation.

An important aspect of the obtained results is the reported motivation of participants to implement the method (76.5%) and the high level of satisfaction with participation in the project (83.6%). Both indicators can be interpreted as factors that strengthen the process of transferring knowledge into practice, in line with the implementation determinants model, where motivation and positive training experiences function as predictors of successful adoption of new solutions. Furthermore, 66.1% of participants declared personal engagement in implementation, indicating the potential for building sustainable changes within professional environments.

An important finding is the high assessment of the potential impact of the Smart Family method on families—over 85% of respondents considered it an effective tool for supporting a healthy lifestyle and increasing self-efficacy, as well as an important factor in encouraging parents to promote healthy behaviors in children. In this context, involving the entire family in the process of changing the home lifestyle, aimed at normalizing body weight, is particularly important and is a key condition for the effectiveness of interventions. This is also confirmed by findings from other programs working with families raising children with obesity [22,23,24].

At the same time, important contextual limitations emerged. Although over half of the respondents (58.7%) considered the timing of the method’s implementation to be appropriate, only 43% believed that their workplace had sufficient resources to implement it effectively. These data indicate that despite high acceptance and individual motivation, structural barriers exist that may limit the practical application of SMART FAMILY. Therefore, effective implementation requires not only participants’ substantive preparation but also the provision of adequate institutional and organizational conditions.

The analysis of barriers to the implementation of the Smart Family method indicates the presence of both social and organizational challenges. The most frequently reported problem was the lack of cooperation from families (87.8%), which underlines the key importance of engaging the entire family in the process of lifestyle change. Such lack of cooperation can significantly limit the program’s effectiveness. One possible reason for this phenomenon is the families’ previous experiences, which may have involved being judged, criticized, or lectured regarding health and lifestyle. These experiences can create barriers and generate resistance to change, reducing willingness to participate in intervention programs. Lack of family engagement in intervention projects, especially in the prevention and treatment of childhood obesity, is a widely observed and well-documented phenomenon in scientific literature [25,26,27]. In this context, the Smart Family method, based on the idea of motivational dialog, appears to address existing needs and barriers. Its main goal is to build cooperation with the family through positive communication, appreciation of strengths, and avoidance of judgment, criticism, or reproach. This approach focuses on pointing out the family’s strengths, recognizing what they are doing well, and identifying areas where they already apply positive practices. Such communication fosters greater motivation, reduces resistance, and builds a positive relationship within the family which can significantly enhance the effectiveness of implemented interventions.

Among the key barriers to the implementation of the SMART FAMILY method, participants indicated a shortage of staff (81.0%), lack of participation in training (78.4%), and limited time to carry out the program (73.2%). In addition, more than two-thirds of respondents reported a lack of materials (70.6%) and insufficient support from supervisors (67.3%) and colleagues (65.7%). These findings highlight the need to provide training resources and institutional support. In response to these challenges, the project organizers ensured continuous support by making materials available on the project website, sending newsletters, offering a free e-learning course, and providing a professional guide, which may support sustainable implementation of the method [16,17,19]. These barriers are a common phenomenon in the implementation of similar initiatives, as confirmed by numerous studies pointing to difficulties related to family engagement, professional uncertainty, changes in professional roles, lack of knowledge and skills, and limited competence in working with caregivers in natural settings [25,26,27].

In summary, the results indicate high motivation and a positive reception of the Smart Family method, while also revealing significant implementation barriers, including lack of experience, staff shortages, limited resources, and insufficient engagement from some families. The findings highlight the need for systematic training of specialists and the provision of institutional support. The methodology of the motivational interview was recognized as a key element in effective work with families. Supported by educational tools and e-learning, the Smart Family program represents an important step toward enhancing professional competencies and strengthening early interventions in the prevention and treatment of childhood obesity.

It should be emphasized that the study did not include long-term follow-up (e.g., 6–12 months post-training), which limits the ability to assess whether professionals actually use the Smart Family tools in lifestyle counseling; future research should incorporate a longitudinal analysis to evaluate the sustainability of changes in professional practice.

## 5. Conclusions

A high level of acceptance and motivation among participants indicates that the SMART FAMILY method has the potential for effective adaptation in Poland. Its implementation requires strengthening specialists’ skills and providing appropriate organizational resources. Overcoming barriers such as lack of experience, limited time, and difficulties in engaging families is crucial to achieve lasting intervention outcomes.

## 6. Study Limitation

This study has several limitations. The research sample consisted of voluntary participants in the SMART FAMILY Program, which may cause a self-selection bias. Data were collected mainly through self-report questionnaires, limiting objectivity. The lack of long-term follow-up prevents conclusions about the durability of the intervention effects. The diversity of participants’ organizational contexts may restrict the generalizability of the findings. Additionally, the absence of analysis of demographic and occupational variables limits understanding of the conditions influencing method implementation.

## Figures and Tables

**Table 1:** Participants’ opinions on the training and the implementation potential of the Smart Family method.

To What Extend do You Agree with the Following Statements?	Completely Disagree *n* (%)	Somewhat Disagree *n* (%)	Neither Agree Nor Disagree *n* (%)	Somewhat Agree *n* (%)	Completely Agree *n* (%)	Total *n* (%)
I am personally interested in the topics covered in this training.	6 (2.5)	9 (3.8)	31 (13.0)	60 (25.2)	132 (55.5)	238 (100)
I am confident that after this training and after becoming better acquainted with the materials, I will be able to implement the Smart Family method.	5 (2.1)	15 (6.3)	47 (19.8)	70 (29.4)	101 (42.4)	238 (100)
I am motivated to implement the Smart Family method.	6 (2.5)	13 (5.5)	37 (15.5)	74 (31.1)	108 (45.4)	238 (100)
The Smart Family method is useful in my work.	17 (7.1)	21 (8.8)	54 (22.7)	45 (18.9)	101 (42.5)	238 (100)
The Smart Family method help promote the lifestyle and self-efficacy of my customers.	2 (0.8)	7 (2.9)	25 (10.5)	60 (25.3)	144 (60.5)	238 (100)
The Smart Family method motivate parents to actively participate and promote their children’s lifestyles.	3 (1.3)	6 (2.5)	26 (10.9)	64 (26.9)	139 (58.4)	238 (100)
The Smart Family method benefit my work community and its operating methods.	12 (5.1)	18 (7.6)	46 (19.4)	66 (27.8)	95 (40.1)	237 (100)
The time is suitable for implementing The Smart Family in my work.	22 (9.3)	31 (13.1)	45 (18.9)	57 (24.1)	82 (34.6)	237 (100)
My work has enough resources to implement the Smart Family.	29 (12.4)	42 (17.8)	63 (26.8)	52 (22.1)	49 (20.9)	235 (100)
I am happy to participate in the implementation of the Smart Family.	5 (2.1)	9 (3.8)	27 (11.4)	55 (23.4)	140 (59.3)	236 (100)
I am committed to implementing the Smart Family.	14 (5.9)	16 (6.8)	50 (21.2)	50 (21.2)	106 (44.9)	236 (100)

**Table 2:** Perceived obstacles to implementing the Smart Family method in participants’ work, *n* (%).

Do You Think the Following Things Can Be an Obstacle to Implementing the Smart Family in Your Work?	Not an Obstacle *n* (%)	Yes, It Can Be an Obstacle *n* (%)	Total *n* (%)
Lack of own education	50 (21.6)	181 (78.4)	231 (100)
Lack of time	62 (26.8)	169 (73.2)	231 (100)
Lack of materials	68 (29.4)	163 (70.6)	231 (100)
Lack of personnel	44 (19.0)	187 (81.0)	231 (100)
Existing routines	84 (36.5)	146 (63.5)	230 (100)
Lack of support from immediate supervisor	74 (32.3)	155 (67.3)	229 (100)
Lack of support from co-workers	79 (34.3)	151 (65.7)	230 (100)
Lack of cooperation with families	28 (12.2)	202 (87.8)	230 (100)

**Table 3:** Participants’ confidence in implementing and acceptance of the Smart Family method in their professional practice, *n* (%).

How Confident Are You Right Now That…	Very Doubtful *n* (%)	Fairly Doubtful *n* (%)	Neither Doubtful Nor Confident *n* (%)	Fairly Confident *n* (%)	Completely Confident *n* (%)	Total *n* (%)
you will be able to change your way of working in accordance with the Smart Family model	10 (4.4)	16 (7.1)	62 (27.6)	62 (27.6)	75 (33.3)	225 (100)
the Smart Family method can be implemented in your work community	15 (6.7)	19 (8.4)	68 (30.2)	56 (24.9)	67 (29.8)	225 (100)
your customers, families and children, like the Smart Family method	21 (9.3)	16 (7.1)	87 (38.7)	45 (20)	56 (24.9)	225 (100)

## Data Availability

The data supporting the findings of this study will be made available to interested researchers upon reasonable request.

## Abbreviations

The following abbreviations are used in this manuscript:

WHO	World Health Organization
COSI	Childhood Obesity Surveillance Initiative
